# AI-powered cognitive remediation for schizophrenia: a psychologist's lens on evidence and scalability in India

**DOI:** 10.3389/fpsyg.2026.1873888

**Published:** 2026-08-05

**Authors:** Drishti Gehlot, Sheetal Musale

**Affiliations:** Department of Psychology, School of Vedic Sciences, MIT Art, Design and Technology University, Pune, India

**Keywords:** artificial intelligence, cognitive remediation, low- and middle-income countries (LMICs), schizophrenia, smartphone interventions, virtual reality

## Abstract

**Background:**

Schizophrenia spectrum disorders (SSDs) are marked by cognitive deficits, functional impairment, and notable treatment gap in India, with lifetime prevalence of 1.41%, current prevalence as 0.42%, and the gap of 72%, added by a severe workforce gap, that is, 0.75 psychiatrists per 100,000 and 0.29 clinical psychologists per 100,000. Scalable tools are required by the psychologists that can fill the substantial urban-rural gap.

**Aim:**

To evaluate how effective, feasible, and clinically applicable AI-powered cognitive remediation is, emphasizing India/LMIC contexts.

**Methods:**

PRISMA-guided umbrella review with narrative synthesis of records published between 2020–2026, identified 48 studies (14 randomized controlled trials (RCTs), 22 reviews, 8 pilots, and 4 bibliometric studies) across PubMed, Scopus, Web of Science. Cognition, functioning, and feasibility, retention, and usability were included as outcome measures.

**Results:**

AI-based AI-powered cognitive remediation showed generally favorable effects. Reported effect sizes ranged from moderate to large across different interventions and outcomes. VR-based social-cognition training showed largest benefits and smartphone-based programs showed promise for working memory and engagement. Hybrid or monitored formats showed generally better retention and usability than fully self-directed interventions. However, the evidence remained heterogeneous, and many findings came from small studies, feasibility work, or reviews of mixed quality.

**Conclusion:**

AI-powered CRT appears to be a promising model for schizophrenia rehabilitation when delivered through psychologist-monitored hybrid models. Such approaches may improve access, support rural and semi-rural service delivery, and help reduce the treatment gap through scalable, culturally adaptable, and technology-enabled care, especially in rural India.

## 1 Introduction and background

According to DSM 5-TR schizophrenia spectrum and other psychotic disorders (SSDs) comes under the category of serious mental health disorders (American Psychiatric Association, 2022). These disorders are characterized by thought content, perceptions, emotions, and behaviors, including schizophrenia, schizoaffective disorders, delusional disorders, and brief psychotic disorders. Cognitive impairments are a primary manifestation of schizophrenia, appearing as precursors to psychosis and setting the stage for future problems in daily functioning (American Psychiatric Association, 2022; Vita et al., 2024).

Worldwide lifetime prevalence of SSDs is 1% (20 cases per 1,000 people); in India, it is 1.41% (0.42% weighted point prevalence), with treatment gap of 72%, thereby representing a substantial unmet mental health burden in India (Gururaj et al., 2016; Hegde et al., 2023). In India, nearly one in seven persons, i.e., 197 million persons, suffer from mental disorders of various kinds, with schizophrenia contributing substantially to the disability-adjusted life years (DALYs) (Gururaj et al., 2016). Cognitive deficits in working memory, attention, executive functions, and social cognition persist even when positive and negative symptoms are controlled. These symptom domains often play a major role in determining the outcome of schizophrenia in terms of the psychosocial functions of the individual (Vita et al., 2024).

These cognitive functions play a leading role in the daily chores of the individual. In a developing country like India, where health infrastructure is lacking and proper social supports are not provided to the mentally ill patients, cognitive burden adds to the economic challenges for individuals, society, and the nation. In India, psychosis peaks at the age of 40–49 years when the educational status of the individual is low (Hegde et al., 2023).

### 1.1 Cognitive remediation and its promise

Cognitive remediation for schizophrenia is a research-backed intervention that enhances cognition and functioning through the involvement of active trained therapists, systematic cognitive exercises integrated with psychiatric rehabilitation to support real-world transfer (Vita et al., 2021). Face-to-face CRT, clinic-based or at home, produces small but clinically significant improvements in attention, memory, problem-solving, and real-world function (Deshpande et al., 2016; Wykes et al., 2011). CRT is thus a “bridge” between biological interventions such as antipsychotic medication and community-based function, addressing gaps that cannot be filled by medication alone (Wykes et al., 2011; Vita et al., 2024).

### 1.2 Workforce and access barriers in India

In India, the scope of CRT application is greatly restricted due to the lack of professional mental health workforce; psychiatrists to the population ratio is merely 0.75 per 100,000, compared to the WHO's recommendation of 3 per 100,000; clinical psychologists are 0.29 per 100,000 (World Bank, 2026). With 70% of population residing in rural/semi-rural areas, access to basic psychiatric treatment is lacking, not to mention CRT (World Health Organization, 2022). CRT is time-consuming and labor-intensive, making its application challenging, especially in the hands of junior staff or community health workers (Deshpande et al., 2016). Furthermore, in rural areas, lack of awareness, irregular follow-ups, and transportation are major issues (Mathur et al., 2024).

### 1.3 Family burden and treatment adherence

The mental health and financial pressures on the caregiver, along with the economic burden of repeated travel to urban centers for treatment, often results in dropout or non-adherence to treatment, thereby increasing the gap between evidence-based treatment outcomes and actual outcomes (Reddy et al., 2017; Mathur et al., 2024). Thus, there are a significant number of people with schizophrenia spectrum disorders in India who are either untreated, undertreated, or treated with medication without the rehabilitative benefits of CRT. Thus, India's mental health scenario seems to be characterized by a significant gap between the urban tertiary treatment centers and the rural primary treatment settings, where the intensity of CRT sessions is too low to bring about significant cognitive benefits (Gururaj et al., 2016; Mathur et al., 2024). This calls for an immediate need for effective, supervised, and contextually adapted CRT to reach out to people with SSDs.

### 1.4 Urban–rural gap in service delivery

Mental health system of India shows an urban–rural divide, with specialized services concentrated in urban tertiary centers and limited continuity of care in rural settings (Mathur et al., 2024; Gururaj et al., 2016). Workforce shortages, poor transportation, infrastructural barriers and fragmented follow-up further constrain access to care in rural communities (Mathur et al., 2024). These barriers point out the need for scalable, primary-care-linked psychosis care.

### 1.5 Scalable psychological and digital solutions

Scaling cognitive remediation therapy (CRT) into rural context requires commitment at a policy level and creativity in terms of models of delivery that can improve access for untreated SSDs (World Bank, 2026). Moreover, Tools, such as Machine Learning (ML) and Artificial Intelligence (AI) are revolutionizing how cognitive remediation therapy is delivered, monitored, and personalized via computerized cognitive training with real-time feedback monitored by psychologists (Chen et al., 2023; McCutcheon et al., 2025).

According to studies from high-income countries, AI assisted CRT yielded medium-to-large effects in cognition, such as VR social cognition and working memory training via smartphones (Chen et al., 2023; Vita et al., 2024). Such technologies are not meant to replace psychologists in India but can extend their reach and enhance the precision of decisions (Deshpande et al., 2016; McCutcheon et al., 2025). Multiple patients can be monitored through psychologist-supported AI-CRT model that have centralized dashboards to track progress, and weaker cognitive domains. Thereby potentially expanding service reach while preserving the clinical integrity of CRT (World Health Organization, 2022; World Bank, 2026). Stepped care models can be integrated with these technologies where complex cases can be taken in regular clinical contact and mild/recovering cases can be given AI-assisted home/community CRT.

### 1.6 The psychologist's lens on AI-powered CRT

From a psychologist's lens, AI-powered CRT is best understood as a **theory-driven and clinically supervised** extension of rehabilitation, rather than as a stand-alone digital product. In this paper, the term “psychologist's lens” refers to these operational criteria: the psychologist retains interpretive authority over AI outputs, clinical responsibility for case formulation, treatment planning, and looking for safety, risk, and cultural adaptation.

Technology can support psychological formulation, structured skill acquisition, adherence monitoring, and relapse-sensitive care. The psychologist retains responsibility for interpretation, feedback, and treatment planning. It can be quite effective for schizophrenia rehabilitation as its main goal is to improve task performance as well as sustained motivation, generalization of gains, and meaningful everyday functioning.

The psychologist's lens is based on clinical principles such as behavioral habit formation, graded exposure to cognitive challenge, and cognitive-behavioral scaffolding. Here, digital exercises, adaptive difficulty, and progress tracking can help patients develop consistent rehabilitation routines, meanwhile app-based data can give psychologists a more objective view of engagement, withdrawal, relapse or cognitive fluctuation between sessions. By doing so, AI-CRT can strengthen the therapeutic alliance by supporting continuity of care instead of replacing human connection.

For India, the psychologist's lens also requires cultural adaptation as well as service-level adaptation. Psychologists can ensure that AI-CRT reflects local language, family structure, idioms of distress, and rural–urban differences in access and digital literacy. This can contribute to the digital psychiatry literature by positioning AI as a psychologically informed tool. Thus, ensuring personalization, monitoring, and scalability, while preserving clinical judgment, and human connection.

### 1.7 Human-in-the-loop and hybrid models

The real value of AI is to aid psychologists to become more efficient and empowered, instead of replacing human connection (Wykes et al., 2011; McCutcheon et al., 2025). Continuous monitoring of performance can help identify when improvement in social cognition or working memory working memory is reducing, initiating adjustments to session duration, frequency, family psychoeducation, and reinforcement techniques (Chen et al., 2023; Vita et al., 2024). This is called “human in the loop,” where the psychologist remains the primary decision maker and AI aids outcome and scalability (Chen et al., 2023). For India's huge SSD population, where comorbid depression, anxiety, and diverse forms of distress are prevalent, psychologists manage collateral psychopathologies and adapt interventions to local literacy, kinship, and caregiving patterns (Reddy et al., 2017).

### 1.8 Monitored AI in the Indian context

Monitored AI methods are particularly pertinent to India, where unregulated digital technologies and commercially driven apps have the potential to spread unchecked, devoid of clinical and ethical considerations (Chen et al., 2023; McCutcheon et al., 2025). The psychologist-led, monitored AI CRT framework ensures that interventions are:

- Built on established CRT interventions such as strategy coaching, generalization to everyday tasks, and massed practice (Wykes et al., 2011; Vita et al., 2024);
- Tailored to local linguistic, cultural, and educational requirements (Deshpande et al., 2016).
- Linked to existing services like primary care mental health initiatives and community-based rehabilitation for feasibility, continuity of care, and reach in India (Mahapatra and Seshadri, 2024).
- Subjected to continuous evaluation for feasibility, safety, and long-term impact on functioning, as opposed to short-term symptom reduction (Chen et al., 2023; Vita et al., 2024).

This is consistent with India's National Mental Health Programe, advocating mental health integration into primary care (Gururaj et al., 2016). Under such a system, psychologists can change from being “frontline interventionists” to “orchestrators” of technology-assisted CRT, becoming evaluators and supervisors (Wykes et al., 2011; McCutcheon et al., 2025).

### 1.9 Scope and contribution of the paper

This paper synthesizes global evidence on AI powered CRT for SSDs from a psychologist's view, focusing on scalable hybrid models to address India's treatment gaps, workforce shortages, and urban-rural differences in India (Chen et al., 2023; McCutcheon et al., 2025). Although there are several review articles on AI applications in SSDs, including prediction, monitoring, and risk stratification, there is little specific on AI powered cognitive rehabilitation tools and their feasibility in low- and middle-income countries (Chen et al., 2023). This review attempts to bridge the gap by examining cognitive and functional outcomes and implementation considerations for psychologists in India (Deshpande et al., 2016).

### 1.10 Practical implications for psychologists

From a conceptual perspective, it positions AI CRT within cognitive behavioral and neurocognitive rehabilitation models, showing how digital technologies can promote CRT components like salience, continuous practice, and real-world transfer via adaptive algorithms and immersive environments (Wykes et al., 2011; Vita et al., 2024). It discusses how smartphone-based and VR-based interventions can be integrated into stepped care models, community rehabilitation, and primary care-based mental health programs, making CRT available for underserved populations (Mahapatra and Seshadri, 2024).

Practically, the paper shows how monitored AI-based models assist psychologists in: managing more patients through real-time analytics and remote supervision (Chen et al., 2023; McCutcheon et al., 2025); modifying CRT to cognitive profiles and functioning (Wykes et al., 2011; Vita et al., 2024). It can enhance collaborations with psychiatrists, primary physicians, and community health workers (Gururaj et al., 2016); and bridging the rural-urban gaps via monitored and technology-based at-home or community CRT (Mahapatra and Seshadri, 2024). In conclusion, this review examines psychologist-driven AI CRT as human-centered innovation to improve therapy and daily lives of SSD patients across India (Deshpande et al., 2016).

## 2 Review of literature

This section reviews recent empirical and review-level work on AI-powered cognitive rehabilitation in schizophrenia spectrum disorders, viewed through a psychologist's lens on evidence (as discussed in section 1.6), mechanisms, and scalability for India. The following literature includes RCTs, systematic reviews, Pilot studies, and bibliometric analyses highlighting AI-powered cognitive remediation, virtual reality, and telerehabilitation.

### 2.1 Landscape and growth of evidence base

With only a moderate but growing number of RCTs and pilot studies, the global evidence base is still young and dominated by narrative and systematic reviews (Khalid et al., 2024; Sun et al., 2025; Du et al., 2025; Yang et al., 2026). A 2025 scoping review by Yang et al. (2026) identified 61 AI-related studies in schizophrenia rehabilitation, most of which were either methodological or descriptive rather than high-power efficacy trials, highlighting that the field is still in early implementation. According to bibliometric studies, cognitive rehabilitation and VR-based research has shown exponential growth since 2016, with over 48% of publications emerging after 2022 (He et al., 2022; Shu et al., 2024; Du et al., 2025).

### 2.2 AI Tools: definitions and significance for CRT

Existing literature shows several AI-related tools and architectures recur with varying levels of clinical maturity. Prediction models for symptom change, relapse risk, or cognitive status are build primarily using machine learning and deep learning. Area-under-the-curve values commonly range between 0.70 and 0.95, suggesting adequate but not definitive discriminative performance (Yang et al., 2026; Wolff, 2025). Reinforcement learning and dynamic difficulty adjustment are included in some VR and robotic systems to personalize task difficulty based on performance. The aim is to balance challenge and frustration during training to improve retention (Lopes et al., 2021; Guzmán et al., 2024; Lee and Kang, 2025).

Delivery platforms such as virtual reality and serious games are used for cognitive and functional tasks combined with biometric feedback or AI-driven adaptivity. Only a subset of systems implements fully automated adaptation or intelligent coaching. Smartphone apps and telerehabilitation platforms uses AI models for adherence verification, automated task progression, or background data analysis. Real-time neurofeedback and conversational agents like robots, represent more experimental applications. They target neural regulation or emotion management rather than core cognitive domains (Markiewicz and Dobrowolska, 2021; Chen et al., 2023; Stefaniak et al., 2026). Overall, AI functions more often as tools for delivery, personalization, and monitoring instead of a completely autonomous therapist. Thus, reinforcing the importance of human oversight in CRT.

### 2.3 Smartphone-based and computerized cognitive training

Studies demonstrated modest but clinically meaningful gains in working memory, attention, and executive function. Along with, high short-term adherence produced by computerized cognitive training (CCT) and smartphone apps (Krzystanek et al., 2020; Aijo and Matsui, 2025; Fathi Azar et al., 2025). MONEO is a smartphone-based intervention. It demonstrated that brief, repetitive pattern-recognition tasks over one year can increase correct response rates and slightly reduce cognitive fatigability relative to assessment-only comparison groups. The completion rates was around 70% in real-world settings (Krzystanek et al., 2020). Although, reduced response-time and the absence of broader functional outcomes in some trials indicate that improved accuracy does not directly translate into faster or more efficient real-life cognition. Feasibility studies of remote or hybrid CCT delivered via tablets or teleplatforms suggest good adherence and pre-post gains in attention and daily functioning. However, these findings derive from small, uncontrolled samples and therefore should be interpreted as preliminary.

### 2.4 Adaptive AI-powered training systems

Machine-learning is used as AI-personalized adaptive training system to modify task difficulty in real time minimizing frustration and maintaining challenges. (Khalid et al., 2024; Khorev et al., 2025; Yang et al., 2026). Yang et al. (2026) explained AI prediction and task-adaptation studies in schizophrenia rehabilitation with area-under-the-curve (AUC) values of 0.70–0.95 for symptom outcomes (Yang et al., 2026). Improve engagement was shown by robot-assisted and VR protocols and allow clinicians to reduce routine training positioning AI as a scalable adjunct to human-led CRT that reduces manual tracking and decision-making burden (Lee and Kang, 2025; Khorev et al., 2025).

### 2.5 Digital therapeutics and remote cognitive remediation in schizophrenia

Systematic reviews of digital health interventions and digital therapeutics for schizophrenia-spectrum disorders suggest that the cognitive benefits are mixed. However, these approaches are generally feasible and well accepted by users. A meta-analysis of 26 randomized controlled trials on digital interventions in psychosis found no clear overall benefit for primary symptom or cognitive outcomes compared with control conditions. Although, the review suggested that programs with structured human support may be more helpful for improving quality of life and some secondary outcomes. It was also noted that many of the included studies had a high risk of bias (Arnautovska et al., 2025).

Small pilot and feasibility studies of remote or digital formats within schizophrenia-specific CRT suggest that therapist-supported or family-assisted models may yield improvements in neurocognitive measures, functioning, and quality of life. But most are single-arm designs or small RCTs without long-term follow-up (Sinha et al., 2023; Singh et al., 2025; Cella et al., 2024). The magnitude of change reported in some pilot trials is substantial. Yet these estimates are likely inflated by small samples, lack of blinding, and regression-to-the-mean effects. Thus, warranting cautious interpretation when extrapolating to routine care.

### 2.6 Hybrid, monitored models in India

Hybrid monitored AI CRT models in Indian psychology involve clinical training along with training using smartphones or computers under the supervision of professionals and correspond to the findings on digital interventions, highlighting the significance of human scaffolding. Indian studies report that CRTs assisted by families and locally designed CRT digital tools could be effective, especially with caregiver and clinician monitoring; however, the research is scarce since it has been done mostly in single-center studies (Kaneko and Keshavan, 2012; Sinha et al., 2023; Singh et al., 2025).

The hybrid models could also bridge the divide between urban tertiary care facilities and rural primary care facilities through their link with initiatives like National Mental Health Program and other district-level services (Mahapatra and Seshadri, 2024). Meanwhile, the digital mental health literature consistently highlights privacy issues, usability, and inequalities in access, so implementing digital interventions at a national level will be impossible without overcoming challenges related to digital illiteracy and infrastructure among others.

## 3 Methodology

### 3.1 Aim

This paper aims to examine global evidence on AI-powered cognitive remediation for schizophrenia spectrum disorders from a psychologist's lens, with a focus on scalable hybrid models addressing India's treatment gaps, workforce shortages, and rural–urban disparities. This paper evaluates various AI-based tools for practical implementation and monitored India's existing mental-health systems by compiling findings from clinical trials, pilot studies, and systematic reviews.

### 3.2 Objectives

The specific objectives are:

- To assess effectiveness of AI-CRT in improving everyday cognitive efficiency in schizophrenia spectrum disorders on the basis of evidences from RCTs and meta-analyses.
- To assess potency, acceptability, and applicability of AI-CRT in low- and middle-income country (LMIC) contexts, focusing on India's workforce limitations and urban–rural discrepancies.
- To propose a hybrid model monitored by psychologists that can be integrated into multi-level care models and community mental health initiatives in India.

### 3.3 Study design

This study used an umbrella review with narrative synthesis based on systematic reviews, meta-analyses, randomized controlled trials, pilot studies, and bibliometric analyses where outcome measures and study characteristics were sufficiently comparable, effect estimates were described descriptively and, when available, in relation to reported pooled estimates. But the review was not intended to produce a new de novo meta-analysis.

### 3.4 Search strategy

Systematic search followed PRISMA 2020 guidelines across various databases, like PubMed, Scopus, Web of Science, Google Scholar from 2020 to 2026. Terms included “artificial intelligence,” “virtual reality,” “computerized cognitive training,” “cognitive remediation,” and “schizophrenia spectrum.” Filters: human studies, adults ≥18 years, English-language; plus, backward/forward citation tracking and gray-literature screening (e.g., conference abstracts, theses). After removing duplicates, 16,400 unique records remained.

### 3.5 Inclusion criteria

Studies were included if they:

- Studied adults (≥18 years) with DSM-5-TR or ICD-11 addressing schizophrenia spectrum disorders, such as schizophrenia, schizoaffective disorder, or other psychotic disorders; mixed cohorts allowed if ≥70% SSDs.
- Used AI/digital CRT targeting ≥2 cognitive domains (e.g., working memory, attention, social cognition, and executive functioning) via adaptive ML, VR, AR, apps, or platforms.
- Compared AI-CRT to traditional interventions or waitlist.
- At least one primary cognitive outcome was reported (e.g., MATRICS, CANTAB, BACS) and one secondary functional outcome (e.g., PSP, SOFAS), as well as feasibility metrics such as retention ( ≤ 20% dropout) and usability (e.g., SUS).
- Used RCTs, quasi-experimental designs, or pre-post pilot studies (*n* ≥ 10) along with quantitative data in peer-reviewed articles.

### 3.6 Exclusion criteria

Studies were excluded if they:

- Used non-AI-based interventions (e.g., pen-and-paper CRT, psychoeducation, or non-computerized psychosocial interventions).
- Included fewer than 70% SSDs or focused primarily on children or adolescents.
- Did not report cognitive or functional outcomes (e.g., symptom-only monitoring).
- Present purely qualitative data, protocols without empirical results, or case studies with fewer than 10 participants.
- Were non-empirical reviews, duplicate publications, or retracted.

### 3.7 Study selection process

**Figure 1:**
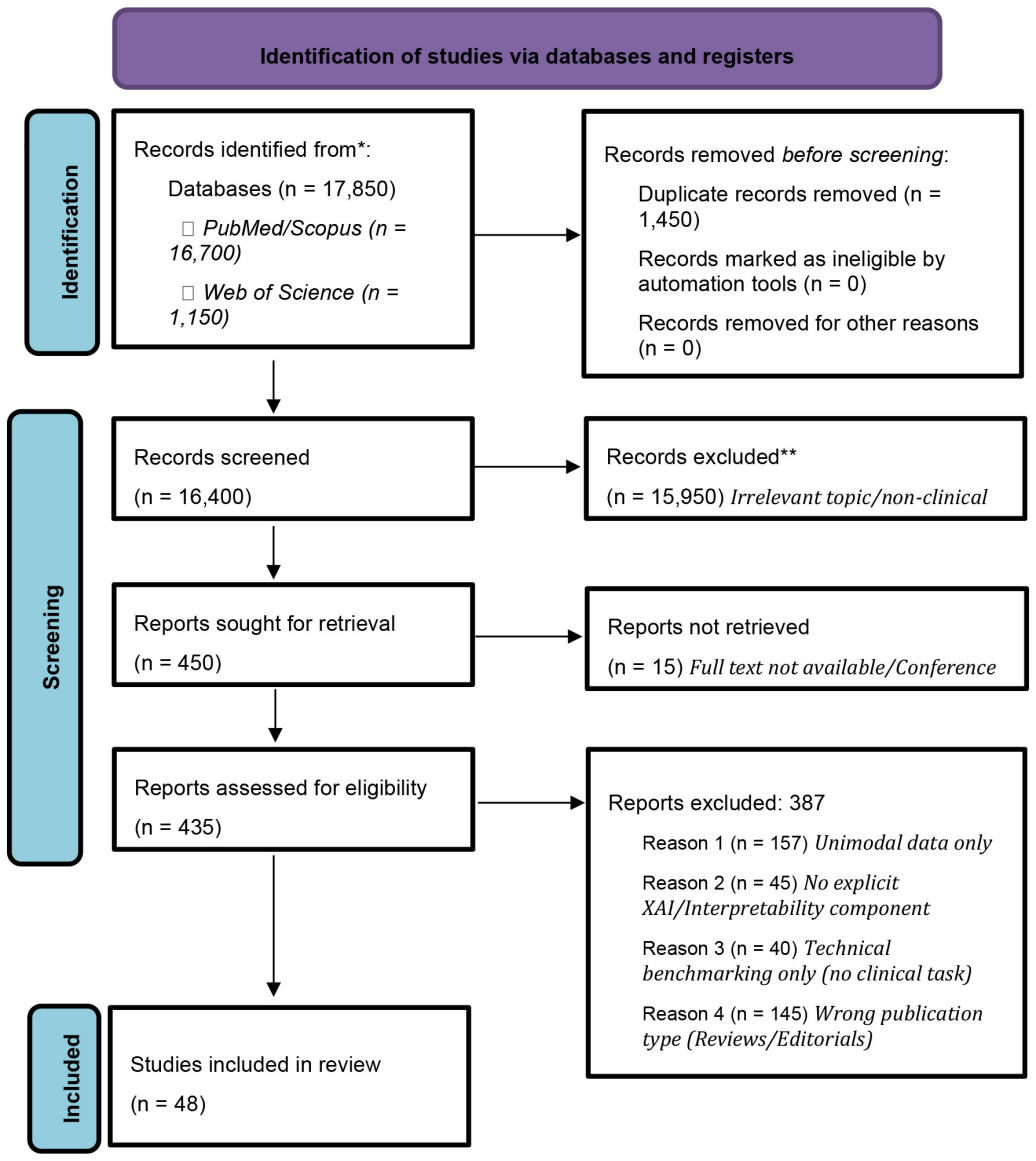
PRISMA 2020 flow diagram.

Screened 16,400 abstracts and titles excluding 15,950 for irrelevance. Full-text assessment of the remaining 450 led to 402 exclusions: Full text not available/Conference (*n* = 15), Non-AI interventions (*n* = 157), < 70% SSD sample or wrong age group (*n* = 45), no cognitive/functional outcome (*n* = 40), wrong publication type/ qualitative-only/protocol-only/case study (*n* = 145). The final sample comprised 48 studies (Figure 1).

### 3.8 Handling double counting and overlap

To reduce the risk of evidence inflation, overlap among primary studies included in multiple systematic reviews was identified and documented. Where multiple reviews covered the same randomized trials, the most recent or most comprehensive synthesis was prioritized. The primary RCTs which were not already represented in the higher-level reviews were summarized separately from rest of the studies. This approach reduced double counting in the narrative synthesis. Although some residual overlap may still be present because umbrella reviews inherently draw on overlapping evidence bases.

### 3.9 Data extraction and risk of bias

Study designs, sample descriptions, interventions, outcomes, and effect sizes were recorded in an excel sheet. Assessment of risk of bias was carried out using RoB 2 for randomized controlled trials and AMSTAR-2 for systematic reviews and meta-analyses. In the current umbrella review, RoB 2 results informed the interpretation of evidence confidence without excluding studies based purely on their quality ratings.

### 3.10 Data synthesis

Data synthesis was performed narratively with domains such as cognitive function, functional performance, and feasibility being considered. Figures and tables were used in summarizing intervention characteristics, outcome trends, and reported effect sizes. As there were methodological differences in terms of the measures, interventions, and dosages, pooled effect sizes were interpreted carefully without considering them as conclusive. Effect size was defined as small (*d* = 0.2), moderate (*d* = 0.5), or large (*d* = 0.8) for interpretation purposes. For interpretive purposes, effect sizes were classified as small (*d* = 0.2), moderate (*d* = 0.5), or large (*d* = 0.8) (estimates derived from primary sources rather than computing new ones).

### 3.11 Ethical considerations

As this review is an umbrella review, based on research that has already been published and is readily available in the public domain, no direct interaction with participants or collection of new data took place; therefore, institutional ethics clearance was not needed. Nonetheless, ethical concerns were addressed when interpreting the findings, especially those pertaining to informed consent for impaired individuals, privacy of electronic and behavior data, algorithmic biases, and the need for human supervision of technology-assisted rehabilitation. Furthermore, the question of whether proper measures for confidentiality, data management, and clinical accountability have been mentioned by included studies was taken into consideration.

### 3.12 Review credibility and reporting quality

The main focus was on transparency, management of overlaps, and careful interpretation, rather than statistical calculations as this is an umbrella review with narrative synthesis. Good quality systematic reviews and RCTs were favored over pilot and descriptive studies.

## 4 Results

The following umbrella review synthesizes 48 studies. It suggests that AI-powered cognitive remediation (AI-CRT) is going from an exploratory phase toward early implementation. Particularly when delivered in hybrid models with professional monitoring. Generally, favorable evidence is present for cognitive benefit but the strength of inference varies substantially by study design, sample size, intervention type, and outcome domain (Khalid et al., 2024; Du et al., 2025; Arnautovska et al., 2025).

### 4.1 Study characteristics and quality

The 48 studies include 14 randomized controlled trials, 22 systematic reviews or meta-analyses, 8 pilot feasibility studies, and 4 bibliometric analyses. The evidence base was therefore weighted toward secondary synthesis and feasibility work. Rather than large confirmatory trials. Double-blind or otherwise controlled RCTs reported statistically significant cognitive gains in several domains. However, effect estimates were heterogeneous. And dependent on intervention format and outcome measure. The findings should be interpreted as promising rather than definitive.

Study samples across the studies were often small to moderate, and many reports lacked external validation, long-term follow-up, or robust comparative designs. This suggests limitations in generalization across illness stage, cultural context, or service setting (Rakitzi et al., 2020; Arnautovska et al., 2025). Accordingly, the present synthesis treats findings from pilot studies and descriptive reviews as exploratory, while giving greater interpretive weight to controlled trials and meta-analytic summaries.

### 4.2 Intervention typology and delivery

Existing literature included intervention such as, virtual reality (VR), computerized cognitive training (CCT), AI/ML-adaptive systems, telerehabilitation platforms, and a smaller set of conversational or robot-assisted tools. Most programs were delivered remotely or at home. Many included at least minimal human support such as weekly clinician check-ins. This suggests that hybrid care models are currently the most practically viable format (Cella et al., 2024; Arnautovska et al., 2025). Doses typically ranged from 20 to 40 h, often delivered 2–5 times per week in 45–60-min sessions, although there was substantial variation across studies (Krzystanek et al., 2020; Fathi Azar et al., 2025).

Within this broad category, VR interventions were commonly used for ecological task practice and structured cognitive or functional training, while CCT platforms emphasized repeated task practice and feedback. AI-adaptive systems more often focused on task personalization, adherence support, or prediction of performance trajectories than on stand-alone remediation of core cognitive deficits (Lee and Kang, 2025; Kim et al., 2025; Yang et al., 2026). These differences are important because outcomes cannot be directly compared across modalities without accounting for delivery intensity, human support, and outcome selection (Table 1).

**Table 1:** Typology of AI-related cognitive remediation interventions.

Tier	Operational definition	Typical features	Examples in this review
**VR**	Immersive or semi-immersive computer-generated environments used to simulate tasks for cognitive, social, or functional training.	Ecological practice, avatar-based interaction, task rehearsal, sometimes sensor or performance feedback.	VR-SCIT, SocialVille, immersive VR training studies.
**CCT**	Structured computerized cognitive exercises delivered on a computer, tablet, or phone with preset tasks and repeated practice.	Repetition, feedback, domain-specific drills, usually non-immersive and rule-based.	CANTAB, RehaCom, COGBRAIN, FACT-style programs.
**AI-adaptive/ML systems**	Systems that use machine learning, reinforcement learning, or algorithmic personalization to adjust difficulty, predict performance, or tailor training.	Dynamic difficulty, adaptive progression, prediction, individualized pathways.	Zenicog^®^, Dr.G-Bot, adaptive VR or RL-based tools.
**Telerehabilitation platforms**	Remote cognitive rehabilitation delivered through web, app, or video-conference platforms, usually with clinician monitoring or hybrid support.	Home-based delivery, remote supervision, adherence tracking, flexible scheduling.	CIRCuiTS™ remote CR, iPad/Teams r-CCT, smartphone-based interventions.
**Robot-assisted tools**	Embodied robotic or conversational agents used to deliver prompts, exercises, or guided interaction for cognitive or social-cognitive rehabilitation.	Voice interaction, social engagement, coaching, emotion regulation, guided tasks.	Terabot, robot-assisted training tools.

### 4.3 Efficacy by cognitive domain

Across domains, AI-CRT and digital CRT were associated with moderate-to-high effect estimates in schizophrenia-specific analyses. But these estimates should be interpreted cautiously because they were derived from interventions with different targets, populations, and comparators. A schizophrenia-focused VR meta-analysis reported a pooled effect of *g* = 0.92. But the underlying studies varied in sample composition, intervention structure, and measurement approach. This indicated that the pooled estimate should be viewed as an upper-range summary rather than a uniform treatment effect (Du et al., 2025).

Working memory and attention were the most consistently improved neurocognitive domains. Whereas executive function showed more variable findings across studies. Reported effect sizes for working-memory outcomes ranged approximately from *d* = 0.40 to 0.95. While executive-function estimates ranged from modest to moderate and were not always statistically robust (Krzystanek et al., 2020; Arnautovska et al., 2025). This pattern suggests that repeated computerized practice may be more reliable for task-specific cognitive efficiency than for broader executive transfer.

### 4.4 VR, CCT, and AI-adaptive outcomes

VR-based interventions produced some of the largest reported gains. Particularly in studies targeting emotion perception, social performance, and ecologically valid functioning. However, these were often small studies with high engagement and limited follow-up. In one pilot RCT, VR-SCIT produced large gains in emotion-perception accuracy and personal/social performance, but the study's sample size and design warrant caution in interpreting the magnitude of benefit (Shen et al., 2022). More broadly, the meta-analytic literature supports a positive but heterogeneous effect for VR-based cognitive interventions, with stronger estimates in schizophrenia-spectrum samples than in mixed clinical groups (Du et al., 2025).

Task performance was moderately improved and adherence was highly achieved by CCTs like MONEO and RehaCom (Moroşan et al., 2025). For instance, according to Krzystanek et al. (2020), there was a notable improvement in correct response rates and completion, while another platform had high usability scores and comparable adherence when using the system without supervision (Krzystanek et al., 2020; Fathi Azar et al., 2025). Such results are promising, but still close to feasibility evidence more than to clinical effectiveness evidence.

Regarding AI- and ML-based systems, they were mostly associated with a prediction ability. The average AUC values ranged from 0.70 to 0.95, which shows that it was possible to classify cases and that such systems could be used effectively as classifiers, but not as treatment tools (Yang et al., 2026; Wolff, 2025). In robot-assisted and telerehabilitation studies, cognitive improvement was found to be small and was similar to therapist-led rehabilitation in terms of efficiency in patients with disorders except schizophrenia (Kim et al., 2025; Lee and Kang, 2025).

### 4.5 Engagement, feasibility, and implementation relevance

Retention and acceptability tended to be positive for the digital and hybrid interventions, and many studies had high rates of participant retention and acceptable usability. Overall, in comparison with the existing CRT services, retention rate in the reviewed reports was somewhat above average, although it is important to take into account possible selection bias, support from researchers and publication bias in favor of viable interventions (Arnautovska et al., 2025; Cella et al., 2024). Engagement can thus be considered one of the relatively stable results from the literature review, even if the results were moderate and limited to particular domains.

Evidence related to potential cost savings and scalability was scarce and should be approached with caution. In one case of therapist-supported remote cognitive remediation, costs were comparable with those incurred by face-to-face therapy, meaning that remote therapy does not appear to increase costs of services, yet there was no proof of any cost savings or economic superiority of remote interventions (Cella et al., 2024). Also, several reviews argued that AI-based and hybrid systems might decrease workload for clinicians, increase reach and help provide wider coverage of services, but again, such claims did not rely on formal health economics (Khorev et al., 2025; Sun et al., 2025).

### 4.6 Relevance to national implementation

Overall, the available evidence seems to lend itself to a conservative implementation-oriented interpretation, whereby AI-CRT seems the most feasible as a complementary solution within hybrid care rather than a standalone one aimed at replacing clinician-delivered interventions. The rationale behind this assumption would be mainly derived from feasibility, acceptability, and non-inferiority evidence rather than from any evidence on a possible nationwide implementation and associated success of this innovation (Cella et al., 2024; Kim et al., 2025; Arnautovska et al., 2025).

As of now, there is no reliable evidence to suggest that cost savings or successful implementation on a national scale have been achieved with the use of AI-CRT in schizophrenia services. Thus, assumptions related to the possible cost savings, scalability, and integration into public mental health services can only be considered testable hypotheses. In other words, hybrid AI-CRT may reduce the burden of clinicians, become more accessible beyond tertiary clinics, and become part of the service pathway when adjusted to the local context of infrastructure, digital literacy, and supervision availability. The latter is especially pertinent to India.

## 5 Discussion

### 5.1 Bridging the rural–urban gap

This review implies that AI-powered CRT can be helpful as an adjunct to schizophrenia rehabilitation instead of becoming a substitute for treatment under the supervision of a professional psychologist. From the results obtained in the reviewed studies, the most robust evidence was found in hybrid and supervised models, whereas the self-directed system revealed weaker results which proves the necessity of human intervention in the process of cognitive therapy (Cella et al., 2024; Arnautovska et al., 2025; Du et al., 2025). In India, this aspect is especially relevant since there is a problem with the uneven distribution of specialists working with schizophrenia patients who live mostly in urban areas leaving rural and semi-urban regions without proper assistance (Gururaj et al., 2016; Hegde et al., 2023).

Therefore, the implementation of the technology in question will be the most beneficial if it is integrated into the healthcare delivery chain rather than used independently as a separate digital tool. Smartphones‘ surveillance technologies, VR-based rehabilitation, and clinicians' dashboard might enable providing structured cognitive therapy in those places where a visit to specialists is complicated but only if these technologies are adapted to local conditions (Khalid et al., 2024; Khorev et al., 2025). At present, the literature supports this as a plausible implementation direction, not as a proven national solution.

### 5.2 Psychologists as clinical supervisors

An AI model driven by the psychologist shows greater support from research compared to the autonomous one. Studies presented in the review show that positive results can be reached through utilization of AI in order to help the practitioner monitor patient practice, adjust complexity and record engagement, whereas the latter remains in charge of the formulation, feedback and treatment process (Kim et al., 2025; Lee and Kang, 2025; Yang et al., 2026). This approach is especially crucial in schizophrenia, where the disorder is typically combined with cognitive impairment, negative symptoms, lack of insight, unstable motivation and treatment resistance.

The use of AI technology can aid the psychologist in summarizing patient progress, noticing problems with adherence, and recognizing patterns of responses which may not be noticeable during standard therapy. However, this information must be seen as an auxiliary one, since the effectiveness of a model highly depends on the data used in its creation (Khalid et al., 2024; Wolff, 2025). The clinical relationship, psychoeducation and contextual analysis play the key role here, especially if the practitioner needs to provide help in comprehending the disorder and its management.

### 5.3 Advantages of AI-CRT

The primary benefit of the AI-CRT approach is not the automation of the therapist role, but personalization. According to the literature, adaptive training, VR-based exercises, and digitally tracked activities allow for better customization of the amount and level of difficulty of practice as compared to static manually developed protocols, which might contribute to the sustainability of engagement and task-related improvements (Fernández-Sotos et al., 2020; Shen et al., 2022; Du et al., 2025; Fathi Azar et al., 2025; Shen and Wu, 2026). Some instruments demonstrated good usability and completion rate as an indicator of the patient tolerance of repetitive digital practice.

The ability to simulate real-life situations is another potential benefit. VR and applications may replicate a realistic context of social interaction and performance of routine tasks, which can be beneficial in terms of rehabilitation in the absence of natural settings. In the case of India, the localization option (usage of the local language, the scenery of a typical village, culturally familiar characters) might add to the relatability of the intervention. However, currently available literature only supports these ideas from a theoretical standpoint rather than empirically proves them.

### 5.4 Indian rural implementation

Implementation for India could plausibly proceed in the form of a hybrid community model with psychologists conducting assessment and formulation, AI-assisted clinical practice modules, and periodic evaluation through PHCs or district-level mental health systems. ASHAs, community health workers, or local nurses can provide assistance with onboarding, navigation of the technology, adherence support, and problem-solving of logistical issues, while the psychologists maintain oversight and manage the risk factors. It is in keeping with the general evidence that low levels of human support increase the effectiveness of retention and usability of digital mental health interventions (Cella et al., 2024; Arnautovska et al., 2025).

The aforementioned framework is potentially compatible with government institutions and the National Mental Health Programme, but the current state of the evidence base does not demonstrate the success of scaling the AI-CRT intervention at a population level in India. As such, the implementation of the intervention at the national level must be considered a phased process of pilot implementation in selected districts, co-design with local stakeholders, modification for language and literacy level, and incorporation into the existing system. It is better-defended than the assumption of success across diverse rural environments.

### 5.5 Ground-level barriers and ethics

The implementation of AI-CRT in rural India is also faced with significant practical difficulties at the ground level. They include poor internet connectivity, unreliable electricity, restricted access to devices, poor digital literacy, and lack of necessary backup infrastructure for prolonged sessions. Cognitive severity, prominent negative symptoms, and treatment resistance can also hinder engagement of patients with app-based or VR-based interventions, especially those which require sustained attention and independent problem solving.

When it comes to ethical issues, informed consent can be more difficult in patients who lack insight or are unfamiliar with digital technology. Privacy becomes important while collecting behavior data through the use of apps. Algorithmic bias is a separate issue since the model trained in other regions will perform worse with rural population, women, elderly, and illiterate population. Apart from infrastructural barriers and digital literacy issues, there might be other problems like, household-related problems in terms of the financial costs of devices, data, maintenance, and time lost at work or while providing care.

### 5.6 Cautious interpretation

Overall, the evidence reviewed here supports a cautious and optimistic interpretation where AI-CRT appears promising tool as a support tool for psychologists, especially when it is personalized, monitored, and embedded in hybrid care pathways. But current findings remain stronger for feasibility, usability, and short-term cognitive change than for durable real-world recovery or national-scale effectiveness (Krzystanek et al., 2020; Shen et al., 2022; Du et al., 2025; Arnautovska et al., 2025). For rural Indian context, the next step is not immediate replacement of conventional care but co-development, local validation, and implementation research in rural and low-resource settings.

### 5.7 Proposed future model

From the available literature, a possible future direction that might emerge for India is the use of a hybrid AI-CRT process headed by the psychologist. Where the clinician handles assessment, formulation, and risk management, while the AI-enabled modules can be employed for practice, monitoring, and customization. The process could be adopted involving primary health centers. Along with the assistance of ASHA workers or community health workers for orientation, follow-ups, device navigation, and adherence. In rural areas, then VR scenes and app-based practices can be culturally tailored using local language content, village scenes, and everyday life situations. However, this is just a hypothesis and needs to be pilot-tested.

## 6 Limitations

### 6.1 Methodological heterogeneity and study quality

The paper includes synthesis of 48 studies, including 14 randomized controlled trials, 22 systematic reviews or meta-analyses, 8 pilot studies, and 4 bibliometric analyses. Although, coverage of the emerging field is strengthened by breadth but it limits formal meta-analyses because of substantial heterogeneity in study design, sample size, intervention format, comparator conditions, and outcome selection. Sample sizes ranged from very small pilot samples to moderate trial samples. While outcome measures varied widely, including MATRICS, CANTAB, MMSE-K, and app-specific indices. Intervention durations also differed considerably across studies, typically spanning 20–40 h delivered over 2–5 sessions per week making direct comparison difficult (Krzystanek et al., 2020; Wykes et al., 2011; Du et al., 2025; Khorev et al., 2025).

### 6.2 Geographic and cultural bias

Most trials were conducted in high-income countries (United States, Europe, Japan, and China), with no Indian or other LMIC-based RCTs, limiting generalizability to India's sociocultural and infrastructural context (Khalid et al., 2024; Khorev et al., 2025). VR and app-based tasks often use western-centric scenarios and language, which may not align with the lived reality of rural, multilingual, or low-literacy Indian patients (Khalid et al., 2024; Thibaudeau et al., 2024). Without culturally adapted case studies, and context-specific design, AI-CRT risks low ecological validity and reduced engagement (Khorev et al., 2025; Yee et al., 2025).

### 6.3 Patient selection and generalizability constraints

The literature largely includes stable, remitted, or outpatient-based schizophrenia-spectrum patients. This excludes many acute-phase, first-episode, and treatment-resistant cases common in India (Vita et al., 2024; Fulford et al., 2025; Gururaj et al., 2016). Participants are also often tech-savvy and relatively high-functioning, which means the study underrepresented marginalized, low-digital-literacy, or severely ill subgroups. For Indian psychologists, this means current evidence best supports AI-CRT as an adjunctive stabilization-phase tool rather than a first-contact or crisis-intervention strategy (Wykes et al., 2011; Vita et al., 2024).

### 6.4 Outcome measurement and follow-up gaps

Cognitive outcomes were reported more frequently than functional or real-world outcomes, and symptom or recovery-oriented measures were less consistently included. Employment, independent living, caregiver burden, and long-term psychosocial functioning were reported in only a small proportion of studies (Arnautovska et al., 2025; Du et al., 2025; Krzystanek et al., 2020; Shen et al., 2022; Vita et al., 2024; Fulford et al., 2025). Follow-up periods were also generally short, with many studies assessing outcomes within 6 months or less. This makes it difficult to determine whether observed gains are durable, transferable to everyday functioning, or clinically meaningful over time.

### 6.5 Technological and accessibility barriers

Implementation of AI-CRT and VR-based systems require stable internet connectivity and relatively powerful devices often unavailable in rural primary health-care centers where internet coverage and smartphone capabilities are less, this can lead to significant infrastructure and access limitations in rural areas in India (Khalid et al., 2024; Khorev et al., 2025; World Bank, 2026). Other factors that create barriers in implementation of AI interventions and retention of patients include high data costs, shared-device use, battery life, and other privacy concerns. (Chivilgina et al., 2021; Khalid et al., 2024). For patients with significant cognitive or motivational impairments, self-guided AI-CRT may be insufficient without clinician-monitored scaffolding, highlighting the need for simplified, culturally sensitive, and human-assisted designs (Wykes et al., 2011; Khorev et al., 2025).

### 6.6 Risk of bias and publication effects

The literature may overestimate benefit because positive findings are more likely to be published, and small-study effects are plausible in an emerging field dominated by feasibility and pilot work. A large proportion of studies report statistically significant outcomes. Some VR-based interventions show very large effect sizes. But these results often come from small or highly selected samples under controlled conditions (Shen et al., 2022; Du et al., 2025; Khorev et al., 2025). The rapid increase in publications after 2022 further raises the possibility of early-stage enthusiasm effects. This means pooled estimates should be interpreted cautiously.

### 6.7 Economic and ethical knowledge gaps

There is limited evidence on cost-effectiveness data for AI-CRT in LMIC settings as most of the data comes from high income bracket and may not fit India's labor-cost structure and service-delivery models (Khalid et al., 2024; Khorev et al., 2025). Quality-adjusted life-year (QALY) analyses and hospitalization-saving models are absent which limit their utility for National Mental Health Programe decisions (Arnautovska et al., 2025; Fulford et al., 2025). Along with this, ethical concerns such as privacy of speech and video data, informed consent in cognitively impaired patients, and bias across caste, region, and language groups remain unexplored (Chivilgina et al., 2021; Khorev et al., 2025; Khalid et al., 2024).

### 6.8 Aggregate impact on conclusions

Overall, the evidence suggests AI-powered CRT is promising but is still in development phase and have moderate-to-large effect sizes (*g* ≈ 0.67–0.92) and high retention (87.6%) documented mainly under controlled, high-income conditions (Krzystanek et al., 2020; Shen et al., 2022; Fathi Azar et al., 2025; Du et al., 2025; Khorev et al., 2025). The findings should be viewed as strong pilot-level evidence justifying well-designed, India-specific trials and implementation studies rather than as proof of ready-to-scale deployment (Khalid et al., 2024; Shu et al., 2024).

## 7 Future directions

### 7.1 India-specific clinical trials

A key priority is to conduct-specific Phase II/III RCTs of AI-powered cognitive remediation (AI-CRT) in individuals with schizophrenia-spectrum populations, including rural and semi-rural groups (Khorev et al., 2025). Smartphone-based computerized cognitive training (CCT) models such as MONEO showed roughly 75% working-memory gains over 1 year providing a strong template, but these must be adapted to Indian linguistic, cultural, and infrastructural contexts (Krzystanek et al., 2020; Khalid et al., 2024; World Bank, 2026). Trials should be done to test hybrid models combining AI-driven training with monitoring by psychologists (Khorev et al., 2025; Arnautovska et al., 2025).

### 7.2 AI Localization and technical adaptations

For AI-CRT to be relevant in India, further work must focus on lived realities of the users. Indian-language-specific NLP models (e.g., Hindi-BERT), Hinglish-compatible conversational agents, and computer-vision systems trained on Indian facial-expression data would lead to more valid outcome and retention (Khalid et al., 2024; Chivilgina et al., 2021; Khorev et al., 2025). Platform that are offline-capable, voice assisted and require low battery usage are more likely to be better suited, particularly in rural settings (Khalid et al., 2024).

### 7.3 Scalable delivery infrastructure

Scalability of AI-powered cognitive remediation will depend on how well it is integrated in India's existing infrastructures (Khalid et al., 2024). Integrating with Ayushman Bharat Digital Mission and WhatsApp-based mini-apps can help in scalability (Khalid et al., 2024). A cascade-training model linking a small group of psychiatrists using AI dashboards, several thousand psychologists supervising hybrid CRT protocols, and a large network of ASHA workers managing onboarding and basic support can harness India's 10-lakh-strong community-health workforce to reach rural populations (Gururaj et al., 2016).

### 7.4 Functional and economic validation

Future research needs to move beyond cognition to measure real-world outcomes that impacts daily functioning such as employment retention, independent-living practices, and family-burden. This can be achieved using locally adapted tools such as MATRICS-India and cultural appropriate PSP variants (Vita et al., 2024; Khorev et al., 2025). Measures such as quality-adjusted life-year (QALY) estimates, cost-effectiveness analyses and reduction in relapses will be important for convincing policymakers to incorporate AI-CRT into National Mental Health Policy-aligned service packages (Arnautovska et al., 2025; Khorev et al., 2025).

### 7.5 Next-generation innovations

Emerging technologies offers promising ways to improve engagement and personalization in CRT. AI-based conversational agents, low-cost EEG-driven neurofeedback, and personalized virtual environments can make interventions more interactive and adaptive to individual needs. For Indian psychologists, these innovations represent opportunities to co-design “human-in-the-loop” systems where AI handles routine tasks and feedback. While clinicians focus on higher-order interpretation and relational care.

### 7.6 Global LMIC generalizability

India's experience with AI-CRT can serve as a practical test-bed for hybrid, low-cost, AI-augmented CRT. Collaboration across multiple centers (e.g., NIMHANS-IIT-CDAC partnerships) can together refine algorithms, edge computer systems, and language-specific NLP models. This openly share protocols, ethical guidelines, and cost-model templates. It will help other LMICs also to build on shared evidence rather than start from scratch (Chivilgina et al., 2021; Khorev et al., 2025).

### 7.7 Regulatory and ethical governance

India must build robust regulatory and ethical frameworks for AI-CRT. These can include end-to-end encryption of speech and video data, proactive testing for caste, region, language-based algorithmic bias, and post-market surveillance dashboards. This can track safety signals and engagement patterns (Khorev et al., 2025). Integrating AI-CRT into Ayushman Bharat structures can support sustainable financing and help protect vulnerable populations from cost- or consent-barriers. For Indian psychologists, active involvement from designing low-literacy-appropriate consent forms to advising on clinician-level alert-thresholds will be essential to ensure that AI-CRT remains rooted in human-centered, and ethical care rather than entirely artificial intelligence (Wykes et al., 2011; Khorev et al., 2025).

## 8 Conclusion

This umbrella review demonstrates that AI-powered cognitive remediation is a promising adjunctive approach for schizophrenia spectrum disorders. The current evidence is stronger for feasibility, acceptability, and short-term cognitive gains than for definitive superiority over conventional CRT. Working memory, engagement, VR-based or personalized digital interventions showed most consistent benefits. Although, effect estimates may vary across populations, comparators, and outcome domains.

Hybrid psychologist-supervised models are more defensible than fully self-directed systems as suggested by the findings in the paper. Human-supported formats generally showed better retention and more practical clinical utility. Purely automated approaches remain less certain in terms of generalizability and long-term impact.

In Indian context, the value of AI-CRT lies in its potential to aid psychologists and extend rehabilitation to under-resourced settings. This can be done through culturally adapted, digitally assisted care. However, this potential depends on local feasibility, digital literacy, infrastructure, ethical safeguards, and sustained clinical oversight rather than on claims of immediate large-scale replacement or cost savings.

In conclusion, AI-powered CRT should be viewed as an evolving, psychologist-led rehabilitation strategy rather than an established standard of care. Future Indian research should prioritize feasibility studies, implementation research, and controlled trials that test whether these tools can be integrated into routine practice while preserving equity, clinical responsibility, and human connection.

## Data Availability

The original contributions presented in the study are included in the article/supplementary material, further inquiries can be directed to the corresponding author.
